# Validation of the Malay Oral Impacts on Daily Performances and Evaluation of Oral Health-Related Quality of Life in a Multi-Ethnic Urban Malaysian Population: A Cross-Sectional Study

**DOI:** 10.3390/ijerph192416944

**Published:** 2022-12-16

**Authors:** Fei Yee Lim, Chui Ling Goo, Wai Keung Leung, Victor Goh

**Affiliations:** 1 Oral Health Division, Ministry of Health, Putrajaya 62590, Malaysia; 2 Faculty of Dentistry, The National University of Malaysia, Kuala Lumpur 50300, Malaysia; 3 Faculty of Dentistry, The University of Hong Kong, Hong Kong SAR, China

**Keywords:** dental caries, ethnicity, Malaysia, malocclusion, oral health, quality of life, racial groups, toothache

## Abstract

Oral Impacts on Daily Performances (OIDP) can be used as a generic or condition-specific oral health-related quality of life (OHRQoL) instrument. It offers different contexts on how dental conditions affect OHRQoL. This cross-sectional study aimed to validate a newly translated Malay OIDP (OIDP-M), compare OHRQoL, decayed, missing, or filled teeth (DMFT) in Malaysians, and investigate factors associated with OHRQoL. A total of 368 Malaysians were surveyed and examined for DMFT. Short-form oral health impact profile-Malaysia [S-OHIP(M)] and OIDP-M were used to measure OHRQoL. The OIDP-M was tested for reliability and validity. DMFT, S-OHIP(M), and OIDP-M between ethnicities were compared. Associations between ethnicity, DMFT, S-OHIP(M), and OIDP-M of Malays and Chinese were evaluated through partial correlation. Malays and Chinese had more filled teeth and DMFT compared with Indians. Malays reported worse OHRQoL through S-OHIP(M). Decayed teeth were positively associated with S-OHIP(M), physical, psychological, social disabilities, and handicap. For OIDP-M, decayed teeth were positively associated with OIDP-M, working, and sleeping. Missing teeth and ethnicity were positively associated with eating and speaking. Filled teeth were negatively associated with cleaning teeth. The OIDP-M was reliable and valid for evaluating OHRQoL. There were differences in DMFT and OHRQoL between ethnicities. Ethnicity affects OHRQoL, where Malays experienced worse OHRQoL due to dental problems.

## 1. Introduction

Oral health-related quality of life (OHRQoL) assessments are used to understand how oral diseases affect each individual’s daily life [1,2,3]. The concept of OHRQoL plays a crucial role when communicating with the lay-population and policymakers [4]. For instance, clinical indicators, such as the decayed, missing, and filled teeth (DMFT) index [5] may only prove meaningful to dental practitioners, whereas the impact of dental caries in terms of inability to eat, rest, or work because of dental pain makes OHRQoL more relatable to the general population [4].

Oral health-related quality of life is a subjective assessment that is strongly tied to individual and environmental characteristics [6,7]. Malaysia is a multi-ethnic country with Malays being the major ethnic group, compromising approximately 69% of the population [8]. Chinese (23%), Indians (7%), and other ethnic groups (1%) make up the rest of the population [8]. Different ethnicities within the same population may have varying perceptions regarding their OHRQoL [9]. In Malaysia, generic measures, such as the short-form oral health impact profile-Malaysia [S-OHIP(M)] [10] has been adapted to evaluate OHRQoL amongst Malay speaking adults. As an alternative to the S-OHIP(M), the Oral Impacts on Daily Performances (OIDP), which can be used as both a generic and condition-specific instrument [11], may prove to be useful in estimating the oral health needs of the adult Malaysian population.

The OIDP was previously translated into the Malay language and validated in a thesis a decade ago [12]. However, there has been no independent, peer-reviewed, or indexed publication of the validation of the Malay OIDP for the general adult population, nor have publications employing the previous Malay OIDP focused on evaluating the minimally important differences (MID) when assessing the impact of specific oral conditions on OHRQoL. As such, to further confirm and improve the applicability of a Malay OIDP in Malaysia, a new translation and validation process, with a slightly larger sample size, and with analyses based on the MID, was conducted [13].

The overall dental health of Malaysians and their utilization of dental care facilities was recently reported in a national survey [14]. However, the impact of dental health on OHRQoL amongst different ethnicities in Malaysia remain unclear. As each ethnicity may view the importance of dental health differently, and experience varying impacts on OHRQoL due to different dental problems, it may be important to compare the dental health status of different ethnicities and associate them with their OHRQoL. Such information may prove useful in planning a more targeted approach for providing governmental aid and social support to promote better dental care utilization in areas where certain ethnicities are more populous [14,15].

The aims of this study were to firstly validate the newly translated Malay language OIDP (OIDP-M); second, to compare the dental health (in terms of DMFT) and OHRQoL between three major ethnicities in an urban Malaysian population employing both the S-OHIP(M) and OIDP-M; and third, to evaluate the association between ethnicity, DMFT, and OHRQoL. The null hypothesis for the second aim was that there were no differences in dental health and OHRQoL between the three major ethnicities in urban Malaysians. The null hypothesis for the third aim was that there were no associations between ethnicity, DMFT, and OHRQoL.

## 2. Materials and Methods

### 2.1. Study Design and Ethics Approval

This was a cross-sectional study divided into 2 parts. The first was a questionnaire survey for OIDP-M validation, and the second was a clinical examination. The study was carried out in accordance with Strengthening the Reporting of Observational Studies in Epidemiology (STROBE) guidelines [16]. The population, exposure, comparator, outcome (PECO) framework [17] was as follows: population included Malays, Chinese, and Indians in urban Malaysia; exposures were decayed, missing, and filled teeth (secondary outcomes and independent variables); comparators were ethnicity (independent variable); and outcomes were S-OHIP(M) and OIDP-M (primary outcomes and dependent variables). Ethics approval was obtained from the Research Ethics Committee, National University of Malaysia (UKM) [UKM PPI/111/8/JEP-2017-550] prior to study commencement.

**Figure 1:**
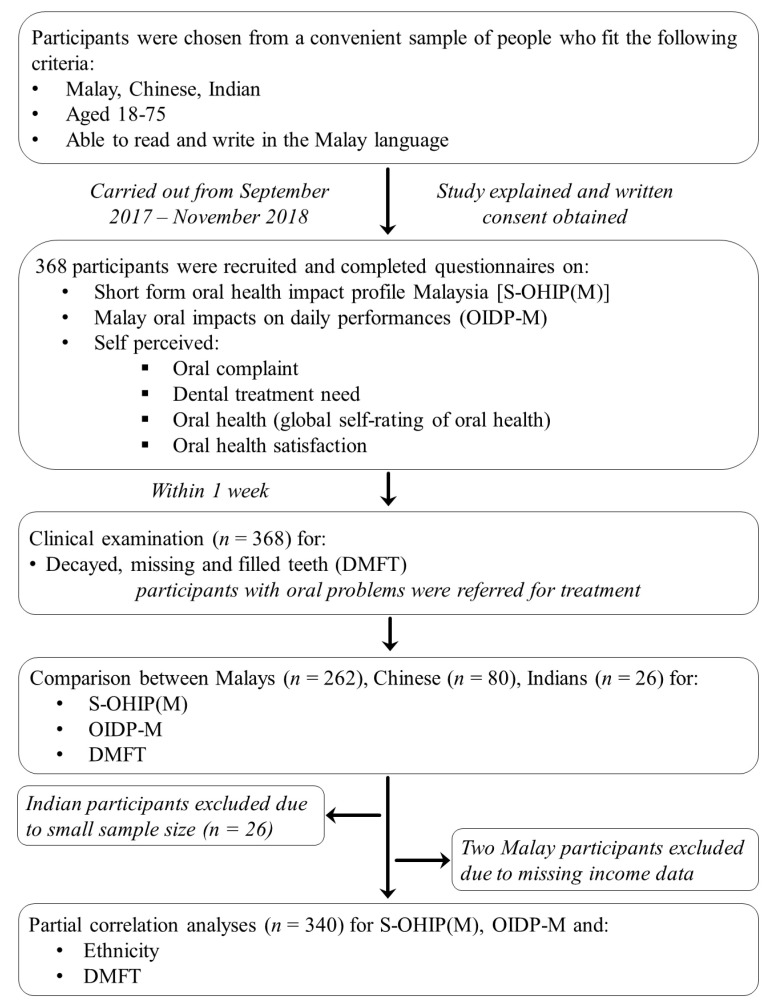
Participant recruitment, assessment, and inclusion for data analyses.

Sample size calculation was based on the following formula: [Z^2^P’(1 − P’)]/d^2^. Where Z was a standard value corresponding to the desired confidence level (Z = 1.96 for 95% confidence interval), P’ was the estimated prevalence of oral impacts (73%) based on a population study in Thailand employing the OIDP [11], and d is precision (5%). This gave an estimated sample size of 303. This sample size was deemed adequate for comparison between the OIDP-M and S-OHIP(M) [18] and validation of the OIDP-M based on previous studies [12]. Subject recruitment was carried out within a 5-mile radius of the UKM Kuala Lumpur campus, including the entire university grounds, the Kuala Lumpur general hospital, surrounding shops, and public facilities. All participants were given a full explanation regarding the purpose of the study. Written consent was obtained from all participants. Participant recruitment is summarized in Figure 1.

### 2.2. Questionnaires

Two questionnaires were used in this study. The first questionnaire was the S-OHIP(M) [10]. The S-OHIP(M) assessed OHRQoL in terms of 7 domains (i.e., functional limitation, physical pain, psychological discomfort, physical disability, psychological disability, social disability, and handicap). Each domain consisted of 2 questions which were assessed on a Likert scale and coded from 0 (never) to 4 (very often).

The second questionnaire was a newly translated Malay Oral Impacts on Daily Performances (OIDP-M). The new OIDP-M translation process is summarized in the Supplementary Materials, while the validation process and clinical examination results are reported in this study. The OIDP-M contained a survey regarding impacts on 8 daily performances: (1) eating and enjoying foods, (2) speaking and pronouncing clearly, (3) cleaning teeth, (4) carrying out major work or social roles, (5) sleeping and relaxing, (6) smiling, laughing, and showing teeth without embarrassment, (7) maintaining a usual emotional state without being irritable, (8) enjoying contact with people. The original OIDP was translated from English into Malay through forward–backward translation and was pre-tested on 60 participants. The final version of the OIDP-M underwent psychometric analysis based on guidelines established by the consensus-based standards for the selection of health status measurement instrument (COSMIN) group [19].

### 2.3. Data Collection

Data collection undertaken through questionnaires included: participants’ demographic data, dental attendance [20], self-perceived oral complaint (yes, no), self-perceived dental treatment need (yes, no), and global self-rating of oral health (GSROH) [21] on a 5-point Likert scale (very poor, poor, neither good nor poor, very good, good), and self-perceived oral health satisfaction on a 3-point Likert scale (dissatisfied, moderately satisfied, satisfied), S-OHIP(M) and OIDP-M. Questionnaires were all self-administrated. These data were used for validation of the OIDP-M and comparison of results between S-OHIP(M) and OIDP-M.

### 2.4. Psychometric Properties Analysis of the OIDP-M

A cross-sectional study was carried out using the finalized OIDP-M for psychometric analyses [22]. Time required to complete the OIDP-M was recorded. Difficulty in understanding and answering the OIDP-M was assessed using a 3-point ordinal scale (easy, moderate, difficult).

#### 2.4.1. Reliability

The OIDP-M was tested for internal reliability and test-retest reliability. Internal reliability indicates the extent to which the OIDP-M items are inter-correlated, or whether they are consistent in assessing the same construct [23]. Internal reliability was determined using Cronbach’s alpha [23,24] based on the performance scores of the first OIDP-M administration. Correlations between each performance were evaluated using inter-item correlation. 

Test-retest reliability refers to the extent to which respondents’ answer to the OIDP-M items remain acceptably consistent across repeated administration [23]. Test-retest reliability in terms of the intraclass correlation coefficient (ICC) was determined from the OIDP-M scores of the first and second administration (Figure S1) from a sample of >100 recalled participants.

#### 2.4.2. Validity

Validity was assessed to ascertain whether the OIDP-M measures what it is intended to measure [24]. Content and face validity were evaluated through studying the items within the measure and relating them to the feedback from the assessed population [25]. Both content and face validity were subjectively evaluated and not statistically tested [25]. Content validity was verified by the expert committee and face validity was assessed through feedback from the pre-test participants. Content validity needed to fulfill 2 criteria, the measure had to be considered valid by experts, and it needed to cover all the required aspects of the concept being measured [25]. Face validity refers to whether an instrument seems as though it would measure what it intends to measure [25]. Face validity was assessed using results from the qualitative interviews that were carried out during the pre-test phase. The participants’ comments were gathered and discussed among the expert committee to achieve a consensus in the OIDP-M development.

Construct validity refers to the ability of an instrument to actually test the theory/hypothesis it was designed to measure [23]. The construct validity of a questionnaire can be assessed by evaluating its association with known variables with which it should be correlated positively or negatively to [23,26]. Construct validity of the OIDP-M was assessed using discriminant validation and convergent validation strategies [24].

Discriminant validity ensures that a measure is able to discriminate between groups of participants with known differences. For this, the ability of OIDP-M to distinguish between groups with different (1) self-perceived oral complaint (Yes/No), and (2) self-perceived dental treatment need (Yes/No), was assessed using the Mann–Whitney U test. Those who had an oral complaint or felt that they needed dental treatment would be more likely to score higher in the OIDP-M.

Criterion validity refers to the ability of a questionnaire to measure how well one measure predicts the outcome of another measure [23,26]. Criterion validity was determined through correlating OIDP-M scores with (1) S-OHIP(M) and (2) GSROH. Although there was no “gold standard” for OHRQoL assessment in Malaysia, the S-OHIP(M) was the most frequently used Malay-language OHRQoL instrument in the country [27,28]. Both the OIDP-M and S-OHIP(M) were evaluated at the same time point for concurrent validity. 

Convergent validation aims to determine if a measurement is related to the variables it attempts to assess. This was determined by correlating (Spearman’s rho) OIDP-M scores with (1) GSROH [21] and (2) self-perceived oral health satisfaction.

### 2.5. Clinical Examination

All participants consented and underwent a clinical examination, which was carried out by a single experienced clinician (FYL). The DMFT index [5] was used to evaluate dental health, as caries and missing teeth were the most common complaints among urban Malaysians [7]. Decayed teeth were determined as a code 3 [localized enamel breakdown (without clinical visual signs of dentinal involvement)] or higher according to the International Caries Detection and Assessment System (ICDAS) [29,30]. Those with dental emergencies were immediately referred for treatment, while those with non-urgent oral problems were given referrals for 6 weeks after completion of the OIDP-M questionnaire survey (Supplementary Information, Figure S1).

### 2.6. Statistical Analysis

The S-OHIP(M) contained 14 items. Each item was given a score using a 5-point Likert scale based on the frequency of experience for each item. The sum of each item’s score generated a total S-OHIP(M) score; a higher S-OHIP(M) score indicated poorer OHRQoL. For OIDP-M, each performance score was determined by multiplying the frequency score with the corresponding severity score. Summation of all performance scores was divided by the maximum possible score and multiplied by 100 to provide a percentage score. A higher S-OHIP(M) or OIDP-M score both indicated poorer OHRQoL. 

The data collected were analyzed using IBM SPSS version 22.0 (SPSS Inc., Chicago, IL, USA). The level of significance was set at 0.05 unless stated otherwise. The Kolmogorov–Smirnov test was employed to test normality of data. Validation of the OIDP-M, analyses of demographic data, DMFT, self-perceived assessments, and condition-specific OIDP-M scores were performed on all participants (*n* = 368). Due to a low sample size for the Indian group, only data from Malays and Chinese were used for correlation analyses. To investigate factors that affected OHRQoL, correlation analyses (controlling for any independent variables that may show initial differences between Malays and Chinese) were carried out with S-OHIP(M) (total and subscales) and OIDP-M (total and subscales) as dependent variables. Independent variables were: (1) ethnicity, (2) decayed teeth, (3) missing teeth, and (4) filled teeth.

Minimally important differences (MID) for condition-specific OIDP-M (CS-OIDP-M) scores from all participants were determined using a distribution-based approach [31]. The standard error of measurement (SEM) was calculated through multiplying the standard deviation of the mean OIDP-M score of the affected group by the square root of one minus the reliability of the OIDP-M index. The SEM value was taken as the MID [31]. Effect size (ES) was calculated using the mean difference in OIDP-M scores between affected and unaffected groups as the numerator, and the pooled standard deviation of the OIDP-M score from both groups as the denominator. The ES was expressed as a ratio and interpreted through benchmark values of small (0.2), moderate (0.5), and large (0.8) effect [32]. The cut-off points at which a specific oral disease/condition was deemed to have impaired OHRQoL was calculated by summation of the MID and the mean OHRQoL score of the unaffected group. Any OHRQoL score equal or above this cut-off point was regarded as being clinically meaningful [31]. The MID for S-OHIP(M) and OIDP-M of participants with no self-perceived oral complaint and no self-perceived dental treatment need was calculated as described above for comparison between the 2 instruments. Participants with no self-perceived oral complaint and no self-perceived dental treatment need but scored more than 0 in the S-OHIP(M) or OIDP-M, were considered the affected groups. Similar participants but with S-OHIP(M) or OIDP-M scores of 0 were taken as the unaffected groups. The MID and ES of selected oral diseases/conditions for S-OHIP(M) scores were not calculated as it was not a condition-specific instrument.

## 3. Results

### 3.1. Socio-Demographic Characteristics

A total of 368 participants [median (interquartile range) age = 28.6 (22.5–37.7)] fulfilled the inclusion criteria and consented to partake in the study. Of the 368 participants evaluated, 262 (71%) were Malays, 80 (22%) were Chinese, and 26 (7%) were Indians. This ratio was generally similar to the current population estimate in Malaysia [8]. Table 1 shows participants’ self-reported socio-demographic data and dental attendance.

### 3.2. OIDP-M Psychometric Properties Analyses

Participants (*n* = 368) took a mean 5.8 ± 2.8 min to complete the OIDP-M. A total of 152 (41.3%) participants found the OIDP-M easy to understand and respond to, 189 (51.4%) found it moderate, while 27 (7.3%) felt it was difficult. Reliability and validity of the OIDP-M was analyzed based on the participants’ response to the OIDP-M and their feedback after OIDP-M administration.

After data collation and cross checking, the following independent variables were recategorized because of small *n* observed in certain subcategories: (i) GSROH categories were collapsed into very poor-neither (very poor, poor, and neither good nor poor) versus good-very good (good and very good); and (ii) self-perceived oral health satisfaction was collapsed into dissatisfied-moderately satisfied versus satisfied.

#### 3.2.1. OIDP-M Reliability

##### Internal Reliability

For internal reliability, the Cronbach’s alpha of OIDP-M was 0.75, which was acceptable [25]. The reported mean inter-item correlation of 0.27 (Supplementary Information, Table S1) was within the acceptable range of 0.15–0.50 [33]. Inverse correlation was found between “speaking” and “sleeping” (Table S1). Inverse correlation and a lower item-total correlation than the cut-off points of 0.15–0.50 (between “speaking” and “emotion”) suggested that “speaking” could be considered for elimination from the OIDP-M. Similarly, inter-item correlations beyond the 0.15–0.50 range were also found between “sleeping” and “smiling”, and “smiling” and “contact” (Table S1). However, correlations between these items and other performances were acceptable. Omission of each item did not improve mean inter-item correlation but resulted in a lower/similar value (range 0.24–0.27). Single omission of every item resulted in reduction/maintaining the original Cronbach’s alpha (Table S2). As such, a decision was made to retain all items in the OIDP-M. Only keeping items that correlated most strongly with each other may be redundant [33]. Moreover, including only such items could create an overly narrow scale [33]. To maintain the originality of the English version of the OIDP [34], which has been used for evaluating OHRQoL in a multitude of studies, a collective decision to retain all performances was made.

##### Test-Retest Reliability

Four weeks after initial administration of the OIDP-M, 200 participants were successfully invited for test-retest. From these 200 participants, 105 (52.5%) participants reported that there were no changes in their oral conditions between the two time points. Only these participants were included in test-retest reliability analysis (Figure S1). Ninety-five participants were excluded either due to new or worsening of existing oral problems or had sought remedies for their oral problems elsewhere after the free clinical examination was provided at the National University of Malaysia. 

The median (interquartile range) age of the 105 test-retest participants was 22.6 (21.6–31.1) years. There were 60 (57.1%) Malays, 33 (31.4%) Chinese, and 12 (11.4%) Indians. Seventy-five (71.4%) were female, and 30 (28.6%) were male. The test-retest reliability of the OIDP-M score was ICC = 0.88 (95% CI 0.83, 0.92).

#### 3.2.2. Validity

##### Content Validity

The content of the OIDP-M was thoroughly discussed to ensure that the items in the OIDP-M were representative of the entire theoretical construct the OIDP-M was designed to assess [11]. The final OIDP-M was scrutinized for content validity and accepted by the expert committee [23].

##### Face Validity

During the pre-test phase, all 60 participants (30 from the first pre-test; 30 from the second pre-test) agreed with the range of oral conditions and daily performances, and listed and reported that they understood the meaning of each item and response in the OIDP-M. Only minor word changes were applied to improve comprehensibility. For the finalized OIDP-M, the majority of participants (*n* = 341, 92.7%) reported that the OIDP-M was easy or moderate to understand and respond to.

##### Criterion Validity

Statistically significant positive correlations between OIDP-M and S-OHIP(M) scores (*r* = 0.62, *p* < 0.001), as well as negative correlations between OIDP-M and GSROH (*r* = −0.41, *p* < 0.001), verified the criterion validity of OIDP-M. The OIDP-M performed in a manner parallel to the S-OHIP(M). As with the S-OHIP(M), OIDP-M was positively correlated with self-perceived oral complaint and self-perceived dental treatment need, and negatively correlated with GSROH and self-perceived oral health satisfaction (Table 2).

##### Construct Validity

Construct validity was assessed through discriminant validity and convergent validity. For discriminant validity, the OIDP-M was correlated with self-perceived oral complaint and self-perceived dental treatment need. Statistically significant differences (*p* < 0.001) in OIDP-M scores were observed between those with a self-perceived oral complaint [3 (0.0–9.0); median (interquartile range)] versus those without [0 (0.0–1.5); median (interquartile range)]. Similarly, statistically significant differences (*p* < 0.001) in OIDP-M scores were also observed between those with a self-perceived dental treatment need [2 (0.0–7.0); median (interquartile range)] versus those without [0 (0.0–0.0); median (interquartile range)] (Table 2).

For convergent validity, the OIDP-M established statistically significant correlations with GSROH (*p* < 0.001) and self-reported oral health satisfaction (*p* < 0.001) (Table 2). From the results, the participants’ OIDP-M scores increased as their GSROH changed from ‘good-very good’ [0 (0.0–0.0), median (interquartile range)] to ‘very poor-neither’ [2 (0.0–7.0), median (interquartile range)], and similarly when their self-perceived oral health satisfaction changed from ‘satisfied’ [0 (0.0–0.0), median (interquartile range)] to ‘dissatisfied-moderately satisfied’ [2 (0.0–8.3), median (interquartile range)]. This supports the concept that oral impacts, GSROH, and self-perceived oral health satisfaction are different but complementary approaches in the assessment of OHRQoL [35]. These observed patterns provide evidence of convergent validity. Such correlations also strengthen the convergent validity of OIDP-M via known-group validation [23], where participants who scored lower in the GSROH and reported lesser self-perceived oral health satisfaction were expected to experience worse OHRQoL.

### 3.3. Participant’s Dental Health Status

All 368 participants attended a clinical examination within a week after OIDP-M and S-OHIP(M) administration. Participants had 29 (27–30) [median (interquartile range)] teeth present. Thirty-seven (10.1%) participants wore dentures, and two (5.4%) were completely edentulous. There were statistically significant differences in the number of filled teeth and DMFT between groups, with Indians scoring lower compared with Malays and Chinese in both parameters (Table 3).

### 3.4. Participants’ OHRQoL in Terms of S-OHIP(M) and OIDP-M

Median (interquartile range) S-OHIP(M) score for all participants (*n* = 368) was 13 (7–19). A comparison between S-OHIP(M) total and domain scores between all three ethnicities are shown in Table 4. There were statistically significant differences in S-OHIP(M) total score, functional limitation, physical pain, psychological discomfort, and psychological disability between groups, with Malays faring slightly worse in all parameters.

Associations between S-OHIP(M) scores and self-perceived oral health assessments for all participants (*n* = 368) are shown in Table 2. The S-OHIP(M) was significantly associated (*p* < 0.001) with all self-perceived oral health assessments. A total of 138 (37.5%) participants reported no self-perceived oral complaint and no self-perceived dental treatment need. As the S-OHIP(M) was positively associated with these two self-perceived oral health assessments, participants with no oral complaint and no dental treatment need should ideally not have any OHRQoL impairments and report an S-OHIP(M) closer to zero. The MID for S-OHIP(M) scores of those with no self-perceived oral complaint and no dental treatment need but with positive S-OHIP(M) scores [*n* = 133, cf. *n* = 5 with no oral complaint, no dental treatment need, zero S-OHIP(M)] was determined at 3.3. As the unaffected group were those with S-OHIP(M) scores of zero, the MID of 3.3 was taken as the minimal cut-off point where the OHRQoL of participants with no self-perceived oral complaint and dental treatment need (*n* = 138) were considered impaired. Any score below 3.3 was considered an error [31]. A total of 98 (71% of 138) participants reported S-OHIP(M) scores of ≥3.3 (mean = 11.0 ± 6.0). Only 40 participants (29% of 138) reported S-OHIP(M) scores <3.3 (mean = 3.0 ± 2.0). 

The results of partial correlation analyses for S-OHIP(M) total and domain scores between Malays and Chinese (*n* = 340) are shown in Table 5. Partial correlation analysis was employed to control for income, gender, and self-perceived dental treatment need due to significant differences between Malays and Chinese [Table 1 and Table S3 (Supplementary Information)]. Total S-OHIP(M) score, physical disability, psychological disability, and social disability were positively correlated with decayed teeth. Handicap was associated with both decayed and filled teeth. The association between handicap and decayed teeth remained significant (r = 0.139, *p* = 0.011) when filled teeth was additionally controlled for. Similarly, the association between handicap and filled teeth remained significant (r = 0.111, *p* = 0.042) when decayed teeth was additionally controlled for.

Median OIDP-M score for all participants (*n* = 368) was 28.6 (22.5–37.7). Comparison between OIDP-M total and performance scores between ethnicities are shown in Table S4 (Supplementary Information). There were no statistically significant differences in OIDP-M total and performance scores between ethnicities.

Association between OIDP-M scores and self-perceived oral health assessments for all subjects are shown in Table 2. OIDP-M was associated with all self-perceived oral health assessments. The MID for OIDP-M scores of those with no self-perceived oral complaint and no dental treatment need but with positive OIDP-M scores (*n* = 25, cf. *n* = 113 with no oral complaint, no dental treatment need, zero OIDP-M) was determined at 1.6. As the unaffected group were those with OIDP-M scores of zero, a score of 1.6 was considered the minimal cut-off point where the OHRQoL of participants with no self-perceived oral complaint and dental treatment need were considered impaired. Only 14 (10.1% of 138) participants reported OIDP-M scores ≥1.6 (mean = 7.6 ± 6.9), while 124 (89.9% of 138) participants reported OIDP-M scores <1.6 (mean = 0.08 ± 0.28).

The results of partial correlation analyses for OIDP-M total and performance scores for Malays and Chinese (*n* = 340) are shown in Table 6. The OIDP-M, carrying out work, and sleeping/relaxing were positively correlated with decayed teeth, while cleaning teeth was negatively correlated with filled teeth. Eating/enjoying food, as well as speaking clearly, were positively correlated with both ethnicity and missing teeth. When additionally controlled for missing teeth, the association with ethnicity for eating/enjoying food and speaking clearly remained statistically significant (*r* = 0.112, *p* = 0.041; and *r* = 0.126, *p* = 0.021, respectively).

### 3.5. Dental Complaints and OIDP-M Condition-Specific Assessment

Self-reported toothache (*n =* 91) and malocclusion (position of teeth) (*n =* 73) were perceived as major causes of negative impacts (chosen from the OIDP-M list; participants may choose ≥1 oral problem). Other oral concerns reported by participants were decay (*n =* 55), swollen gums (*n =* 35), tooth loss (*n =* 28), sensitive teeth (*n* = 23), bleeding gums (*n* = 17), oral ulcer/spot (*n* = 17), color of teeth (*n* = 13), loose/ill-fitting denture (*n* = 13), improper filling or crown (*n* = 13), and others (*n* = 48).

To assess the suitability of the new OIDP-M as a condition-specific tool, self-reported toothache and malocclusion were chosen as they were the most frequent oral complaints. A total of 28 (7.6%) participants had their OIDP-M scores affected by self-reported toothache only, and 22 (6.0%) by self-reported malocclusion only. The mean CS-OIDP-M scores calculated from these participants were: toothache only = 5.6 ± 4.8, and malocclusion only = 7.5 ± 9.7. Participants without any oral complaints (*n* = 247, 173 Malays, 53 Chinese, 21 Indians) with mean OIDP-M of 2.6 ± 5.9 were used as the reference/unaffected group to compare against those who complained of toothache only and malocclusion only. The CS-OIDP-M MID score for participants who complained of toothache only was 2.4 with moderate ES (0.51), while for the malocclusion only group, the MID was 4.8 with large ES (0.83). For the toothache only group, 13 (46.4%) participants had CS-OIDP-M scores that were 2.4 points above those without oral complaints. Their mean CS-OIDP-M score was 9.8 ± 3.9. Twelve (92.3%) were Malays, and one (7.7%) was Chinese. For malocclusion only, five (22.7%) participants had CS-OIDP-M scores that were 4.8 points above those without oral complaints. Their mean CS-OIDP-M score was 23.6 ± 6.8. Three (60%) were Malays, and two (40%) were Chinese.

## 4. Discussion

### 4.1. OIDP-M Translation and Psychometric Properties Analyses

The OIDP-M preserved the overall content and concept of the English OIDP via expert committee review and accounted for the views of the multi-ethnic population in Malaysia via pre-testing. The OIDP-M was closely associated with S-OHIP(M) and discriminated appropriately in the expected direction with those reporting positive self-perceived oral complaint, positive dental treatment need, worse oral health (via GSROH), and worse oral health satisfaction scoring higher in the OIDP-M. This verified the construct and criterion validity. It was also easily understood and accepted by the evaluated participants, confirming face validity. Internal and test-retest reliability were within an acceptable range. These results support the use of OIDP-M as an instrument for evaluating OHRQoL in Malaysia. The recruitment of participants in this study included all three major ethnicities who could read and speak the Malay language, allowing better generalizability of OIDP-M as an OHRQoL instrument in a multi-ethnic country such as Malaysia.

### 4.2. Dental Health Status and OHRQOL

The DMFT index was used to evaluate clinical dental health. In general, the number of decayed and missing teeth amongst all participants were lower than filled teeth, which might be due to the positive outcome of easier access to both public and private dental services in an urban area [36]. Statistically significant differences in the number of filled teeth between Malays versus Indians, and Chinese versus Indians, were observed. Malays and Chinese also had significantly higher DMFT scores compared with Indians. A previous study reported similar results where Malays and Chinese had more filled teeth compared with Indians [37]. This may be a reflection of how different ethnicities perceive the importance of oral health care and utilize dental services in Malaysia [37]. Hence, the null hypothesis that there are no differences in dental health between ethnicities was rejected.

Based on the total scores of both S-OHIP(M) and OIDP-M among the 260/80 Malay/Chinese participants, a higher number of decayed teeth were associated with worse general OHRQoL. From the S-OHIP(M), it can be observed that discomfort and pain from carious teeth was associated with physical, psychological, and social disabilities, as well as handicap. Indeed, having pain from decayed teeth can hinder one from performing simple daily activities, such as eating and working [38]. Toothache and esthetic concerns from carious teeth may even escalate to a point where one may shy away from social interactions, which could possibly lead to missed opportunities in the workplace and their private lives [39,40]. The handicap domain of S-OHIP(M) was positively associated with the number of filled teeth. Participants may have felt that having filled teeth was an indication of poor dental health or some form of oral impediment and responded as such.

From the OIDP-M, decayed teeth were associated with disruptions in one’s ability to carry out work, and to sleep or relax. Pain from decayed teeth can reduce work productivity and disrupt one’s daily schedule due to loss of focus and the need to visit a dentist for treatment [38,39]. In the United States of America, over 300 million school or working hours were lost annually due to oral problems such as toothache [41].

A negative correlation was found between cleaning teeth and filled teeth. Having food stuck between teeth due to possible decay was frequently reported by subjects from an urban Malaysian population [7]. The inverse relationship between cleaning teeth and filled teeth suggests that restored teeth may be less plaque retentive and are easier to clean on a daily basis.

Eating food and speaking clearly were positively associated with ethnicity and missing teeth, with Malays being slightly worse off compared with Chinese in both performances. Indeed, it is unavoidable that tooth loss would directly impact oral functions, such as eating and speaking [3]. The impact of tooth loss on the ability to consume food was reported in a Malaysian community made up of largely Malays who exhibited poorer nutritional status and worse OHRQoL due to missing teeth [42]. The effect of tooth loss, especially the anterior teeth, on speech disturbances was also highlighted in a recent systematic review [43], and can be a daily hassle, especially amongst studying/working young adults who need to speak confidently in society [44].

In short, ethnicity, dental health status, and OHRQoL present significant correlations with each other, and the null hypothesis that there were no associations between ethnicity, DMFT, and OHRQoL was rejected.

### 4.3. Participants’ OHRQoL in Terms of S-OHIP(M) and OIDP-M

Within the limits of this study, there were differences in OHRQoL amongst the three major ethnicities in urban Malaysia. Malay participants reported worse OHRQoL in terms of S-OHIP(M) total score, psychological discomfort, and psychological disability compared with Chinese and Indians. Malays fared worse in terms of functional limitation and physical pain compared with Indians, while Chinese experienced more physical pain compared with Indians. Such observations may be due to the psychological profile of Malays [45] who were prone to exhibit the social emotion of shyness and were easily affected by the opinion of others [46]. Malays were perhaps more mindful of their dental conditions and experienced more psychosocial impacts due to dental problems when compared with Chinese and Indians. It has been reported that subjects from lower income groups in urban Malaysia, which consisted of mostly Malays, tend to experience frequent psychological discomfort and higher self-perceived dental treatment needs due to their oral conditions [7]. Differences in reports of functional limitation and physical pain between Malays and Indians, and in physical pain between Chinese and Indians may be attributed to the differences in pain threshold between groups [47].

When Malays and Chinese were compared, ethnicity was not correlated with S-OHIP(M) or any of its subdomains. However, when the OIDP-M was employed, ethnicity was positively associated with eating and speaking. Such observations may be explained by the fact that the OIDP-M was designed to detect only the worst impacts on daily activities, while the S-OHIP(M) captures more general and less severe OHRQoL impairments. Within the current group of participants, ethnicity appears to impair the worst domains of OHRQoL, as detected by the OIDP-M. Taken together, these observations suggest that ethnicity does affect the OHRQoL of those examined, with Malays experiencing a slightly worse OHRQoL than Chinese and Indians. The null hypothesis that there are no differences in OHRQoL between ethnicities was therefore rejected. With this in mind, the OHRQoL of different ethnicities should be taken into consideration when planning oral health care and education in a multi-cultural environment [48]. This will ensure that any treatment rendered can fulfill both the functional and psychological needs of the patients involved.

### 4.4. Dental Complaints and OIDP-M Condition-Specific Assessment

One of the advantages of the OIDP-M is that specific oral conditions are identified as the cause of impact on each daily performance assessed. When only one oral condition is reported, the OIDP-M can be used as a condition-specific measure, and determination of the MID for that oral condition can be undertaken. The MID provides a good indication of whether an observed difference in OHRQoL score between groups was meaningful [31]. The MID value indicates what was probably a measurement error. Therefore, any changes or differences smaller than the MID are presumed to be an error, while any differences equal to or above the MID value were seen as important from the participants’ perspectives [31]. Toothache and malocclusion were major complaints among the evaluated participants. Only data from those who stated that they were impacted by toothache or malocclusion were used for MID determination. In the context of this study, the difference in mean OIDP-M scores between those who complained of toothache only (*n* = 28) and those with no oral complaints (*n* = 247) was three points, while the ES of this difference was moderate at 0.51. The difference in mean OIDP-M scores between groups exceeded the MID (2.4), which was considered to be clinically meaningful. Thirteen participants felt that their OHRQoL was badly affected by toothache only and reported CS-OIDP-M scores ≥2.4 points above the mean OIDP-M score of those without any oral complaint (2.6 points). Similarly, the difference in mean OIDP-M scores between those who complained of malocclusion only (*n* = 22) and those with no oral complaints was 4.9 points, while the ES of this difference (0.83) was large. The difference in mean OIDP-M scores between groups exceeded the MID (4.8), and this was also taken to be clinically meaningful. Five participants were affected by self-reported malocclusion and reported CS-OIDP-M scores ≥4.8 points (MID) above those with no oral complaints. The majority of these two groups of participants were Malays. 

To date, there are no guidelines to determine whether an individual with a specific OHRQoL outcome pattern is mildly, moderately, or severely affected by an oral disease/condition [49]. The calculation of the MID and determination of cut-off points enabled better interpretation of the acquired OHRQoL data. Such interpretation of data was crucial, as statistical significance does not provide key information about the research question, which is to appreciate whether differences between groups were truly meaningful from a patient’s perspective [31]. The results here exemplify the use of OIDP-M as a condition-specific measure, bringing more focus towards oral diseases/conditions that impact one’s OHRQoL so that proper interventions may be carried out to treat the patient and not just the disease.

### 4.5. Comparison between S-OHIP(M) and OIDP-M

The S-OHIP(M) and OIDP-M vary in their content and present different aspects of OHRQoL. A comparison of results from the S-OHIP(M) and OIDP-M was necessary to determine the appropriateness and applicability of each questionnaire in assessing the OHRQoL of a multi-ethnic population. The S-OHIP(M) considers the frequency of different oral impacts, overlooking the actual effect such impacts have on daily activities [35]. This may sometimes result in over-reporting of impacts, even though patients have no actual self-perceived oral problems [35]. On the other hand, the OIDP-M tends to report fewer impacts as it has a narrower focus, and only takes into account the ultimate impacts, which correspond to “disability” and “handicap” as described by Locker’s model [50]. Differences between the two questionnaires can be seen in the proportions of patients who reported no self-perceived oral complaint and no self-perceived dental treatment need (*n* = 138), but still reported OHRQoL impairments with scores above the respective cut-off points (or MID in this case). A total of 98 (71% of 138) participants reported OHRQoL impairments above the cut-off point through the S-OHIP(M), while only 14 (10.1% of 138) participants reported OIDP-M scores above the cut-off point. The S-OHIP(M) was more sensitive at detecting OHRQoL impacts, while OIDP-M revealed only the worst effects.

Using both the S-OHIP(M) and OIDP-M in population studies not only helped detect general impacts of oral conditions/diseases on OHRQoL, but also allowed for a more detailed assessment of how specific conditions/diseases affected the daily lives of those surveyed. The beneficial effect of assessing OHRQoL with both S-OHIP(M) and OIDP-M was observed when trying to understand how decayed teeth actually affected the current cohort. Although total S-OHIP(M), physical disability, psychological disability, social disability, and handicap were impaired by decayed teeth in general, such results do not specify how pain from decayed teeth had actually affected participants’ daily function. From the OIDP-M, it can be seen that participants’ ability to work and sleep/relax was significantly associated with tooth decay, giving us better insight into how dental problems, such as carious teeth, had affected their daily lives. 

In short, the S-OHIP(M) is suitable for large-scale epidemiological studies [35] due to its sensitivity towards less severe impacts [51], allowing larger samples of participants with impacts to be detected. This can help exemplify the importance of addressing oral problems as part of improving general health [52]. The OIDP-M allows self-report of oral diseases/conditions that have negatively affected one’s daily life, providing clinicians better focus when diagnosing and formulating treatment plans for their patients. The condition-specific attribute of the OIDP-M allows for the determination of the MID for known oral diseases/conditions, giving light to specific impacts from a single oral disease/condition on OHRQoL [53]. Within the limits of this study, the differences between the two instruments appear to complement each other and provided a deeper understanding on how dental problems can affect OHRQoL.

## 5. Limitations

Certain limitations must be acknowledged. A cross-sectional study did not allow evaluation of the responsiveness of the OIDP-M. This should be conducted in future clinical trials employing the OIDP-M before and after specific treatment. The participants were conveniently recruited within an urban area, which may not be nationally representative. However, the dental disease level of previous participants surveyed within such urban areas were reported to be similar to the national prevalence [54]. The number of Indian participants was low and therefore excluded from the partial correlation analysis. Future studies with a larger sample size are needed to elucidate the differences in OHRQoL between all three major ethnicities in Malaysia. Another limitation was that the median age of the participants, 28.6 (22.5–37.7), was relatively young, with 70.3% of participants having received tertiary education, and were mostly females (65.5%). This was different from the national norm, where 52% of the population had secondary education and 48% were females [55]. However, education level was not correlated (Spearman’s rho) with OIDP-M (*r =* 0.008, *p* = 0.877) and S-OHIP(M) (*r* = −0.28, *p* = 0.593). Similarly, gender did not influence OHRQoL perception in terms of OIDP-M (Spearman’s rho, *r* < 0.001, *p* = 0.993) and S-OHIP(M) (Spearman’s rho, *r =* 0.020, *p* = 0.702) scores. Moreover, the median age of the evaluated participants was similar to the Malaysian population median age of 28.6 years in 2018 [8]. Other oral conditions, such as periodontal disease, were not assessed. Periodontitis exerts negative impacts on OHRQoL [45,56] and should be evaluated in future research, using a condition-specific approach to determine factors that affect the daily lives of the study population.

## 6. Conclusions

The current translated and validated OIDP-M was found to have acceptable internal and test-retest reliability, good content, construct, and criterion validity and was interpretable for evaluating OHRQoL amongst Malay-speaking adults.

There were differences in dental health status and OHRQoL perceptions between ethnicities. Within the limits of this study, Malays reported worse OHRQoL in terms of S-OHIP(M) total and domain scores when compared with Chinese and Indians. However, these differences were not captured by the OIDP-M. Based on the OIDP-M, when Malays and Chinese were compared, ethnicity was associated with impairments in eating food and speaking clearly, with Malays feeling slightly more impacts compared with Chinese.

Malays and Chinese had more filled teeth and higher DMFT compared with Indians. The presence of specific dental problems, such as decayed teeth, contributed to OHRQoL deteriorations as evaluated by both the S-OHIP(M) and OIDP-M. Toothache and malocclusion were the most frequent oral complaints and exerted negative impacts on OHRQoL among the surveyed participants.

Both S-OHIP(M) and OIDP-M were useful instruments to evaluate OHRQoL among urban Malaysians. Due to the synergistic effect of both OHRQoL instruments, when assessing the OHRQoL of a multi-ethnic Malay speaking society, the use of both S-OHIP(M) and OIDP-M should be considered for a more comprehensive picture of how oral conditions/diseases impact OHRQoL.

## Figures and Tables

**Table 1:** Participants’ self-reported socio-demographic data and dental attendance (*n* = 368).

	Malay (1) (*n* = 262)	Chinese (2) (*n* = 80)	Indian (3) (*n* = 26)	*p*	Post Hoc Analysis
**Age**					
Median (interquartile range)	30 (23.4–37.6)	23.6 (22.1–43.3)	22.5 (21.7–34.5)	0.012 ^a^	(1) > (3)
Mean ± standard deviation	32.5 ± 10.8	32.3 ± 13.8	30.0 ± 14.4		
**Gender**				≤0.011 ^b^	(2) > (1), (3)
Male	84 (32.1)	38 (47.5)	5 (19.2)		
Female	178 (67.9)	42 (52.5)	21 (80.8)		
**Educational level**				0.080 ^b^	
No formal/primary education	3 (1.2)	4 (5)	0 (0)		
Secondary education	68 (26.0)	28 (35)	6 (23.1)		
Tertiary education	191 (72.9)	48 (60)	20 (76.9)		
**Monthly income** ^c^				≤0.010 ^b^	(3) > (2) > (1)
≤RM999	87 (33.2)	53 (66.3)	19 (73.1)		
RM1000-RM2999	94 (35.9)	9 (11.3)	7 (26.9)		
≥RM3000	79 (30.2)	18 (22.4)	0		
Not willing to disclose	2 (0.7)				
**Smoking status**					
Smoker	24 (9.2)	3 (3.8)	1 (3.8)	0.211 ^b^	
Non-smoker	238 (90.8)	77 (96.2)	25 (96.2)		
**Dental care** ^d^				0.386 ^b^	
Symptomatic dental attender	190 (72.5)	54 (67.5)	16 (61.5)		
Regular dental attender	72 (27.5)	26 (32.5)	10 (38.5)		

Values are shown as *n* unless stated otherwise; percentage in parenthesis; statistical significance set at *p* < 0.016; ^a^ Kruskal–Wallis test; ^b^ Chi-square test; ^c^ Malaysian median monthly income in Ringgit Malaysia (RM) for year 2019 = RM2415; ^d^ symptomatic dental attender was defined as those who claimed that they went to the dentist occasionally or only when in trouble, while regular dental attender was defined as those who stated that they went to the dentist regularly for check-ups.

**Table 2:** Comparison and associations between S-OHIP(M) or OIDP-M scores and self-perceived oral health assessments (*n* = 368).

		S-OHIP (M)				OIDP-M ^a^			
				vs. Self-Assessment				vs. Self-Assessment	
	*n* (%)	Med (Q1–Q3)	*p*1 ^b^	r	*p*2 ^c^	Med (Q1–Q3)	*p*3 ^b^	r	*p*4 ^c^
**Self-perceived oral complaint**			<0.001	0.384	<0.001		<0.001	0.37	<0.001
Yes	121 (32.9)	18 (12.5–23.5)				3 (0.0–9.0)			
No	247 (67.1)	10 (4.0–16.0)				0 (0.0–1.5)			
**Self-perceived dental treatment need**			<0.001	0.45	<0.001		<0.001	0.42	<0.001
Yes	219 (59.5)	7 (3.0–13.0)				2 (0.0–7.0)			
No	149 (40.5)	16 (10.0–23.0)				0 (0.0–0.0)			
**Global self-rating of oral health**			<0.001	−0.49	<0.001		<0.001	−0.41	<0.001
Very poor-neither	221 (60.1)	16 (11.0–23.0)				2 (0.0–7.0)			
Good-very good	147 (39.9)	7 (3.0–12.0)				0 (0.0–0.0)			
**Self-perceived oral health satisfaction**			<0.001	−0.43	<0.001		<0.001	−0.37	<0.001
Dissatisfied-moderately satisfied	201 (54.6)	16 (11.0–23.0)				2 (0.0–8.3)			
Satisfied	167 (45.4)	8 (4.0–14.0)				0 (0.0–1.0)			

Med (Q1–Q3) = median (interquartile range); ^a^ same data set used for evaluation of construct/criterion validity for OIDP-M validation; ^b^ Mann–Whitney U test; ^c^ Spearman’s correlation.

**Table 3:** Comparison of decayed, missing, filled teeth, and total DMFT [median (interquartile range)] between ethnic groups (*n* = 368).

Assessed Parameters	Malay (1) (*n* = 262)	Chinese (2) (*n* = 80)	Indian (3) (*n* = 26)	*p* ^a^	Pairwise Comparison
Decayed teeth (D)	0 (0–1)	0 (0–1)	0 (0–0)	0.102	
Missing teeth (M)	0 (0–2)	0 (0–1)	0 (0–1.5)	0.423	
Filled teeth (F)	2 (0–5)	2 (0–4)	0 (0–1.25)	≤0.007	(1), (2) > (3)
Total DMFT	4 (1–8)	3 (1–7)	1 (0–3.5)	≤0.027	(1), (2) > (3)

^a^ Kruskal–Wallis test with pairwise comparison.

**Table 4:** Comparison of S-OHIP(M) total and domain scores [median (inter-quartile range)] between ethnic groups (*n* = 368).

Assessed Parameters	Malay (1) (*n* = 262)	Chinese (2) (*n* = 80)	Indian (3) (*n* = 26)	*p* ^a^	Pairwise Comparison
S-OHIP(M)	14 (7–21)	11 (5–17.5)	7 (2.75–17)	≤0.023	(1) > (2), (3)
Functional limitation	2 (1–4)	2 (0.25–3)	1 (0–2.25)	0.012	(1) > (3)
Physical pain	3 (1.75–4)	2 (1–3)	1 (1–2)	≤0.009	(1), (2) > (3)
Psychological discomfort	3.5 (2–5)	3 (2–4)	2 (1–3.25)	≤0.012	(1) > (2), (3)
Physical disability	2 (0–3)	1.5 (0–3)	0 (0–3)	0.171	
Psychological disability	1 (0–3)	0 (0–2)	0 (0–2)	≤0.028	(1) > (2), (3)
Social disability	0 (0–2)	0 (0–1)	0 (0–1.25)	0.084	
Handicap	1 (0–3)	1 (0–2)	0 (0–2)	0.099	

^a^ Kruskal–Wallis test with pairwise comparison.

**Table 5:** Partial correlation analyses of S-OHIP(M) total and domain scores for Malay or Chinese ethnicity; decayed, missing, or filled teeth (*n* = 340) ^a^.

	Ethnicity ^b^		Decayed Teeth		Missing Teeth		Filled Teeth	
Assessed Parameters	r	*p*	r	*p*	r	*p*	r	*p*
S-OHIP(M)	−0.044	0.420	0.159	0.003	0.039	0.473	0.009	0.873
Functional limitation	−0.010	0.860	0.106	0.052	0.063	0.245	−0.086	0.114
Physical pain	−0.004	0.941	0043	0.428	0.034	0.536	0.014	0.805
Psychological discomfort	−0.099	0.070	0.072	0.186	−0.037	0.496	0.050	0.358
Physical disability	0.007	0.898	0.136	0.012	0.105	0.054	0.028	0.614
Psychological disability	−0.100	0.067	0.206	<0.001	−0.016	0.774	−0.037	0.494
Social disability	−0.020	0.712	0.138	0.011	−0.013	0.813	−0.059	0.284
Handicap	−0.004	0.947	0.137	0.012	0.057	0.299	0.109	0.045

^a^ Analyses controlled for gender, income, and self-perceived dental treatment needs; two Malay participants were excluded from analyses due to missing income data; ^b^ Malays as reference group.

**Table 6:** Partial correlation analyses of OIDP-M total and performance scores for Malay or Chinese ethnicity; decayed, missing, or filled teeth (*n* = 340) ^a^.

	Ethnicity ^b^		Decayed Teeth		Missing Teeth		Filled Teeth	
Assessed Parameters	r	*p*	r	*p*	r	*p*	r	*p*
OIDP-M	0.070	0.198	0.138	0.011	0.100	0.066	−0.057	0.294
Eating/enjoying food	0.118	0.030	0.052	0.339	0.174	0.001	−0.094	0.085
Speaking clearly	0.132	0.015	0.014	0.793	0.166	0.002	−0.061	0.261
Cleaning teeth	−0.023	0.672	0.033	0.543	0.012	0.831	−0.114	0.037
Carrying out work	−0.005	0.933	0.174	0.001	0.015	0.784	−0.053	0.335
Sleeping/relaxing	0.057	0.299	0.261	<0.001	0.037	0.494	−0.016	0.767
Smiling/laughing	0.050	0.357	0.021	0.704	0.017	0.762	0.006	0.908
Maintain emotional state	0.037	0.493	0.101	0.063	−0.011	0.846	0.027	0.627
Enjoying contact	−0.019	0.723	0.093	0.090	0.058	0.287	0.045	0.415

^a^ Analyses controlled for gender, income, and self-perceived dental treatment needs; two Malay participants were excluded from analyses due to missing income data: ^b^ Malays as reference group.

## Data Availability

The data presented in this study are available on request from the corresponding author.

## Supplementary Materials

The following supporting information can be downloaded at: https://www.mdpi.com/article/10.3390/ijerph192416944/s1, Figure S1. Participants’ recruitment, assessment, and recall. Table S1. Evaluation of OIDP-M^a^ internal reliability: matrix of inter-item correlations (*n* = 368). Table S2. Evaluation of internal reliability: corrected item-total correlation and Cronbach’s alpha if item deleted (*n* = 368). Table S3. Comparison of self-perceived oral health assessments according to ethnicities (*n* = 368). Table S4. Comparison of OIDP-M total and performance scores [median (interquartile range)] between ethnic groups. References [57,58,59,60] are cited in Supplementary Materials.
