# Supplementary material for: Validation of the Malay Oral Impacts on Daily Performances and Evaluation of Oral Health-Related Quality of Life in a Multi-Ethnic Urban Malaysian Population: A Cross-Sectional Study
Source: Int J Environ Res Public Health. 2022 Dec 16;19(24):16944. doi: 10.3390/ijerph192416944 (PMC9779849; doi:10.3390/ijerph192416944)
Supplement: Supplementary file 1 [file ijerph-19-16944-s001.zip › ijerph-1994447-supplementary.pdf]

SUPPLEMENTARY INFORMATION TO ORIGINAL ARTICLE

Validation of the Malay Oral Impacts on Daily Performances and Evaluation of Oral Health-Related Quality of Life in a Multi-ethnic Urban Malaysian Population: A Cross-sectional Study.

Fei Yee Lim, Chui Ling Goo, Wai Keung Leung, and Victor Goh

1. Translation of the Malay Oral Impacts on Daily Performances (OIDP-M)

When a questionnaire is to be applied to a different culture, language, and country, the questionnaire needs to be translated and culturally adapted [57]. As such, a thorough translation and validation process for Malay Oral Impacts on Daily Performances (OIDP-M) has been carried out and reported in a thesis [13]. To aid comprehension of the current report, an essential summary of the translation of the OIDP-M is included in this supplement.

2. Materials and Methods

2.1. Study Design and Ethics Approval

The translation and validation process involves two parts [22]. First, the original OIDP [11] was translated into the Malay language. Following this, the psychometric properties of the newly translated OIDP-M was evaluated through a convenient sample, cross-sectional study [22]. The validation process and results are reported in the main text. Ethics approval was obtained from the Ethical Committee, The National University of Malaysia (UKM) [UKM PPI/111/8/JEP-2017-550]. Sample size calculation has been described in the main text. Participant recruitment for OIDP-M validation is summarized in Figure S1.

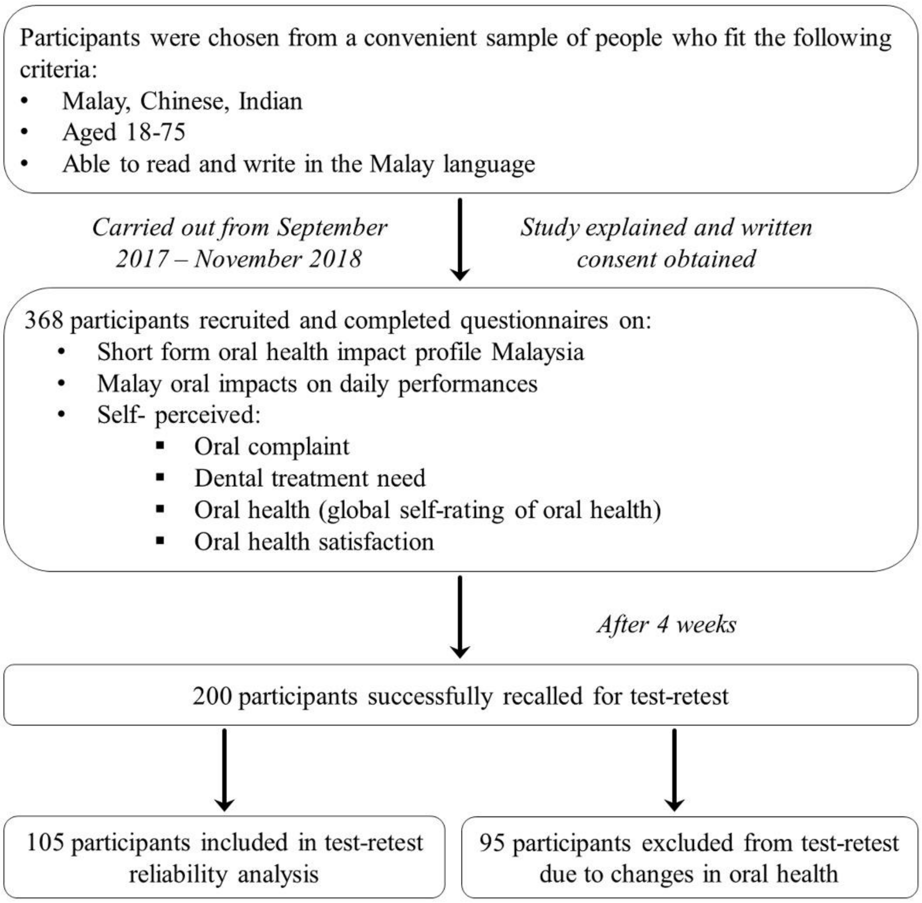

Figure S1. Participants' recruitment, assessment, and recall.

2.2. Translation and Validation of the OIDP-M

The translation and validation of OIDP-M [13] follows the 'consensus-based standards for the selection of health status measurement instrument' (COSMIN) guidelines [58] and adheres in part, to the protocol described previously [59]. However, the responsiveness of the OIDP-M was not evaluated.

2.2.1. Forward and backward translation of the OIDP

The English OIDP [11] contained a survey regarding impacts upon eight daily performances: 1) eating and enjoying foods, 2) speaking and pronouncing clearly, 3) cleaning teeth, 4) carrying out major work or social role, 5) sleeping and relaxing, 6) smiling, laughing, and showing teeth without embarrassment, 7) maintaining a usual emotional state without being irritable, 8) enjoying contact with people. The OIDP translation was carried out in stages as described below.

2.2.1.1. Stage I: Forward translation

Forward translation of the OIDP from English to Malay was done by two independent translators (T1 and T2) who were of different academic profiles and background and were proficient in

the Malay and English languages. T1 was a member of the Malaysia Translators Association, who had no professional dental and oral-health related quality of life (OHRQoL) knowledge, while T2 was a licensed ethnic-Malay dentist who had knowledge on the concepts of OHRQoL. A written report was prepared by each translator and additional comments to highlight challenging phrases or uncertainties were noted.

2.2.1.2. Stage II: Synthesis of the translation

The two translators (T1 and T2) and a moderator (FYL) synthesized the result of the translations. Issues encountered by all involved were resolved by reaching a consensus to produce one common translation (T-12).

2.2.1.3. Stage III: Backward translation

Backward translation of the T-12 version into a new English version was done by two back-translators. These were, BT1: a faculty staff from the School of Language Studies and Linguistics, Faculty of Social Sciences and Humanities, The UKM; BT2: a lay person with tertiary education (without dental/medical background). Both BT1 and BT2 were proficient in both the Malay and English languages and had never come across the original English OIDP.

2.2.1.4. Stage IV: Expert committee evaluation

An expert committee comprised of oral health professionals (FYL, CLG, VG) and the translators (T1, T2, BT1, BT2) were set up. They reviewed the original and back-translated English OIDP and scrutinized all Malay translations to reach a consensus on any discrepancies. Collective decisions were made to ensure semantic, experiential, and conceptual equivalence as well as the syntax and logic. A draft-OIDP-M questionnaire was developed.

Semantic equivalence is equivalence in the meaning of words. Experiential equivalence refers to experiences, or connections to Malaysians’ daily lives. Conceptual equivalence refers to the validity of the concept explored, and how these concepts and terminologies are used relate to events experienced by people in Malaysia. To ensure cultural adaptation of the OIDP-M, the situations depicted in the instrument were ensured to fit the Malaysian cultural context.

2.2.1.5. Stage V: Pre-testing

For the first pre-test (pre-test 1), the draft-OIDP-M was self-administered on 30 participants (20 Malays, 8 Chinese, 2 Indians), who were conveniently recruited from those who visited UKM Kuala Lumpur campus from September 2017 to November 2017 [23]. A qualitative interview was conducted

after each of the participants had completed the questionnaire. Each participant's opinion regarding the daily performances and oral conditions provided, understanding of each item and their corresponding responses, and difficulties in interpreting the items, were recorded. Comments and suggestions from the participants were discussed and assessed by an expert committee [22].

After wording modifications according to the Malay Language Board and Library (Dewan Bahasa dan Pustaka) [60] recommendation, the questionnaire was re-tested (pre-test 2) on another 30 participants (20 Malays, 8 Chinese, 2 Indians) who were recruited from people visiting UKM from January 2018 to February 2018 [23]. The same qualitative interview was conducted, and they were asked the same questions and were invited to comment on the individual daily performances surveyed as well as the syntax and logic of the entire draft-OIDP-M. After the interviews, all 30 participants expressed that they could accept the questionnaire as it is. As such, no further suggestions were offered by them. Based on these results, the expert committee decided to adopt the revised draft-OIDP-M as the finalized OIDP-M to be used in the study.

3. Results

The draft-OIDP-M was pre-tested twice on a total of 60 participants (30 for pre-test 1 and 30 for pre-test 2) with a mean age of 27.8 ± 6.8 years. They consisted of 40 (66.7%) Malays, 16 (26.7%) Chinese, and 4 (6.7%) Indians. Forty-one (68.3%) were female, while 19 (31.7%) were male. All draft-OIDP-M tested participants reported that they understood the meaning of each item and response. No missing item was found. Although minor corrections to the wordings were carried out, no other oral condition or daily performance were suggested to be added to the OIDP-M. The expert committee thoroughly reviewed all translated and amended OIDP-M and reached a consensus on semantic, experiential, and conceptual equivalences as well as syntax and logic of the final OIDP-M.

4. Conclusion

The current translated and validated OIDP-M was found to have acceptable internal and test-retest reliability, good content, construct, and criterion validity and was interpretable for evaluating OHRQoL amongst Malay-speaking adults. This tool was also successfully applied in evaluating OHRQoL in an urban multi-ethnic community (main text). Further research employing the use of the OIDP-M as both a generic and condition-specific tool is needed to widen its use among Malay-speaking populations. Clinical research employing the OIDP-M before and after specific treatment can be carried out to evaluate the responsiveness of the OIDP-M.

Table S1. Evaluation of OIDP-M[a] internal reliability: matrix of inter-item correlations (*n* = 368)

Performance	Eating	Speaking	Cleaning	Working	Sleeping	Smiling	Emotion	Contact
Eating	1.00							
Speaking	.22	1.00						
Cleaning	.42	.28	1.00					
Working	.30	.08	.44	1.00				
Sleeping	.27	-.03	.23	.27	1.00			
Smiling	.38	.24	.21	.20	.10	1.00		
Emotion	.29	.02	.20	.17	.39	.29	1.00	
Contact	.38	.19	.21	.42	.19	.66	.41	1.00

Pearson's correlation coefficient was employed; Mean inter-item correlation = 0.27

[a]OIDP eight daily performances - *Eating*: eating and enjoying foods; *Speaking*: speaking and pronouncing clearly; *Cleaning*: cleaning teeth; *Working*: carrying out major work or social role; *Sleeping*: sleeping and relaxing; *Smiling*: smiling, laughing, and showing teeth without embarrassment; *Emotion*: maintaining a usual emotional state without being irritable; *Contact*: enjoying contact with people.

Table S2. Evaluation of internal reliability: corrected item-total correlation and Cronbach's alpha if item deleted (n = 368)

Performance	Corrected item-total correlation	Cronbach's Alpha if item deleted
Eating and enjoying foods	0.54	0.70
Speaking and pronouncing clearly	0.26	0.75
Cleaning teeth	0.45	0.71
Carrying out major work or social role	0.45	0.72
Sleeping and relaxing	0.32	0.74
Smiling and showing teeth without embarrassment	0.51	0.71
Maintaining a usual emotional state without being irritable	0.41	0.73
Enjoying contact with people	0.61	0.68

Table S3. Comparison of self-perceived oral health assessments according to ethnicities (n = 368)

	**Malay (1) (n = 262)**	**Chinese (2) (n = 80)**	**Indian (3) (n = 26)**	p^a	**Post hoc analysis**
**Self-perceived oral complaint**					
Yes	89 (34)	27 (34)	5 (19)	0.307	
No	173 (66)	53 (66)	21 (81)		
**Self-perceived dental treatment need**					
Yes	172 (66)	37 (46)	10 (38)	≤ 0.006	(1) > (2), (3)
No	90 (34)	43 (54)	16 (62)		
**Global self-rating of oral health**					
Very poor-neither	168 (64)	40 (50.0)	13 (50.0)	0.043	
Good-very good	94 (36)	40 (50.0)	13 (50.0)		
**Self-perceived oral health satisfaction**					
Dissatisfied-moderately satisfied	152 (58)	38 (48)	11 (42)	0.108	
Satisfied	110 (42)	42 (52)	15 (58)		

Values are shown as n; percentage in parenthesis

[a]Chi square test, statistical significance set at $p < 0.016$

Table S4. Comparison of OIDP-M total and performance scores [median (interquartile range)] between ethnic groups

Assessed parameters[a]	Malay (*n* = 262)	Chinese (*n* = 80)	Indian (*n* = 26)	p^b
OIDP-M	0.5 (0 – 5.125)	0 (0 – 5.5)	0 (0 – 4.25)	0.663
Eating/enjoying food	2 (1 - 2)	2 (1 - 2)	2 (1.75 - 2)	0.255
Speaking clearly	2 (2 - 2)	2 (2 - 2)	2 (2 - 2)	0.829
Cleaning teeth	2 (2 - 2)	2 (2 - 2)	2 (2 - 2)	0.159
Carrying out work	2 (2 - 2)	2 (2 - 2)	2 (2 - 2)	0.238
Sleeping/relaxing	2 (2 - 2)	2 (2 - 2)	2 (2 - 2)	0.869
Smiling/laughing	2 (2 - 2)	2 (2 - 2)	2 (2 - 2)	0.973
Maintain emotional state	2 (2 - 2)	2 (2 - 2)	2 (2 - 2)	0.572
Enjoying contact	2 (2 - 2)	2 (2 - 2)	2 (2 - 2)	0.607

[a]OIDP eight daily performances: Eating and enjoying foods; Speaking and pronouncing clearly; Cleaning teeth; Carrying out major work or social role; Sleeping and relaxing; Smiling, laughing, and showing teeth without embarrassment; Maintaining a usual emotional state without being irritable; Enjoying contact with people; [b]Kruskal-Wallis test with pairwise comparison.

References

Continued from main text

57. Beaton, D.E.; Bombardier, C.; Guillemin, F.; Ferraz, M.B. Guidelines for the process of cross-cultural adaptation of self-report measures. *Spine.* **2000**, 25, 3186-3191.
58. Mokkink, L.B.; Prinsen, C.A.; Bouter, L.M.; Vet, H.C.; Terwee, C.B. The consensus-based standards for the selection of health measurement instruments (COSMIN) and how to select an outcome measurement instrument. *Braz J. Phys Ther.* **2016**, 20, 105-113.
59. Ng, S.K.; Stouthard, M.E.; Keung Leung, W. Validation of a Chinese version of the dental anxiety inventory. *Commun. Dent. Oral. Epidemiol.* **2005**, 33, 107-114.
60. Dewan Bahasa dan Pustaka. Available online: http://lamanweb.dbp.gov.my (accessed on 19th July 2017).

Impak Oral ke atas Perlakuan Harian

Malay Oral Impacts on Daily Performances (OIDP-M)

ARAHAN

<u>**AKTIVITI / TINGKAH LAKU** harian</u>

a. Makan dan menikmati makanan

b. Bercakap dan menyebut perkataan dengan jelas

c. Memberus gigi

d. Menjalankan kerja penting atau peranan masyarakat

e. Tidur dan bersenang-senang

f. Senyum, ketawa dan menunjukkan gigi tanpa rasa malu/segan

g. Mengekalkan keadaan emosi biasa tanpa rasa terganggu

h. Menyukai berinteraksi dengan orang lain

<u>**Sila menjawab soalan-soalan berikut pada Jadual jawapan di muka surat 4, 5 dan 6.**</u>
Bagi setiap **AKTIVITI / TINGKAH LAKU** di atas,

Soalan 1 (S1)
Dalam masa 6 bulan yang lepas, adakah anda menghadapi apa-apa kesukaran untuk… **AKTIVITI / TINGKAH LAKU**... disebabkan masalah dengan mulut, gigi atau gigi palsu anda?
(Sila pilih jawapan anda dengan menandakan satu petak di dalam lajur S**1**)
Jika jawapan anda ialah **"ya"** di **S1** – sila beralih ke **S2.**
Jika jawapan anda ialah **"tidak"** di **S1 –** sila beralih ke aktiviti/ tingkah laku yang berikutnya.

Soalan 2 (S2)
Pernahkah anda menghadapi kesukaran untuk ... **AKTIVITI/TINGKAH LAKU**... ini secara kerap/berkala dalam tempoh enam (6) bulan yang lepas atau hanya untuk suatu tempoh/seketika?
(Sila pilih jawapan anda dengan menandakan satu petak di dalam lajur **S2**)
Jika jawapan anda ialah **"secara kerap/ berkala"** di **S2** – sila beralih ke **S3.**
Jika jawapan anda ialah **"hanya untuk suatu tempoh/ seketika"** di **S2 –** sila beralih ke **S4**.

Soalan 3 (S3)
Jawab soalan ini hanya jika anda telah menjawab **"secara kerap/ berkala"** di **S2.** Dalam tempoh enam (6) bulan yang lepas, berapa kerap anda menghadapi kesukaran untuk…**AKTIVITI / TINGKAH LAKU**... ini?
(Sila pilih jawapan anda dengan menandakan satu petak di dalam lajur **S3**)

Soalan 4 (S4)
Jawab soalan ini hanya jika anda telah menjawab **"hanya untuk suatu tempoh/seketika"** di **S2.** Dalam tempoh enam (6) bulan yang lepas, sejauh manakah anda telah menghadapi kesukaran untuk...**AKTIVITI / TINGKAH LAKU**... ini**?**
(Sila pilih jawapan anda dengan menandakan satu petak di dalam lajur **S4**)

Soalan 5 (S5)
Dengan menggunakan skala dari 0 hingga 5, dengan 0 menandakan tiada kesan manakala 5 menandakan kesan yang sangat teruk, betapa kuatnya kesan kesukaran untuk...**AKTIVITI / TINGKAH LAKU**... ini terhadap kehidupan seharian anda?
(Sila pilih jawapan anda dengan menandakan satu petak di dalam lajur **S5** dan beralih ke **S6**)

Soalan 6 (S6)
Manakah antara sebab-sebab dalam Jadual 1 telah menyebabkan kesukaran dalam … **AKTIVITI / TINGKAH LAKU**...?
(Sila pilih jawapan anda dengan meletakkan kod jawapan di dalam lajur **S6**)

Jadual 1: Penyebab

	**Kod Jawapan**		**Kod Jawapan**
Sakit gigi	1	Gangguan deria rasa	14
Kehilangan gigi	2	Mulut kering	15
Gigi goyang/ longgar	3	Gusi berdarah	16
Reput (gigi berlubang)	4	Gusi bengkak (gusi bernanah)	17
Gigi pecah/patah	5	Paras gusi menurun	18
Gigi sensitif	6	Karang gigi	19
Warna gigi	7	Bunyi ‘klik’ pada sendi rahang	20
Bentuk atau saiz gigi	8	Sakit pada sendi rahang	21
Posisi gigi (misalnya tidak lurus atau jongang, jarang)	9	Kesukaran membuka mulut dengan luas	22
Ketidaknormalan pada mulut atau muka (misalnya bibir sumbing, lelangit sumbing)	10	Gigi palsu yang longgar	23
Ulser atau tompok pada mulut	11	Tampalan atau korona gigi yang tidak baik (misalnya pecah, warna tidak bersesuaian)	24
Rasa pedih pada mulut	12	Sebab-sebab lain? (Sila nyatakan)	88
Nafas berbau	13	Tidak pasti	99

Jadual jawapan

	Kesukaran untuk Aktiviti/ Tingkah laku berikut ...	S1: ya/ tidak	S2: Jika ya, berapa kerap	S3: Jika secara kerap/berkala, berapa kerap	S4: Jika hanya untuk suatu tempoh/seketika, berapa hari semuanya	S5: Kesan terhadap kehidupan seharian	S6: Penyebab (kod jawapan)
**a**	**Makan dan menikmati makanan**	1☐ ya<br>2☐ tidak	1☐ secara kerap/ berkala<br>2☐ hanya untuk suatu tempoh/seketika	1☐ kurang daripada sekali dalam sebulan<br>2☐ 1-2 kali dalam sebulan<br>3☐ 1-2 kali dalam seminggu<br>4☐ 3-4 kali dalam seminggu<br>5☐ setiap hari atau hampir setiap hari<br>9☐ tidak pasti	1☐ sehingga 5 hari<br>2☐ sehingga 15 hari<br>3☐ sehingga 30 hari<br>4☐ sehingga 3 bulan<br>5☐ lebih daripada 3 bulan<br>9☐ tidak pasti	0☐ tiada kesan<br>1☐ kesan yang sangat kecil<br>2☐ kesan kecil<br>3☐ kesan sederhana<br>4☐ kesan teruk<br>5☐ kesan yang sangat teruk<br>9☐ tidak pasti	
**b**	**Bercakap dan menyebut perkataan dengan jelas**	1☐ ya<br>2☐ tidak	1☐ secara kerap/ berkala<br>2☐ hanya untuk suatu tempoh/seketika	1☐ kurang daripada sekali dalam sebulan<br>2☐ 1-2 kali dalam sebulan<br>3☐ 1-2 kali dalam seminggu<br>4☐ 3-4 kali dalam seminggu<br>5☐ setiap hari atau hampir setiap hari<br>9☐ tidak pasti	1☐ sehingga 5 hari<br>2☐ sehingga 15 hari<br>3☐ sehingga 30 hari<br>4☐ sehingga 3 bulan<br>5☐ lebih daripada 3 bulan<br>9☐ tidak pasti	0☐ tiada kesan<br>1☐ kesan yang sangat kecil<br>2☐ kesan kecil<br>3☐ kesan sederhana<br>4☐ kesan teruk<br>5☐ kesan yang sangat teruk<br>9☐ tidak pasti	

Sambungan Jadual jawapan…

	Kesukaran untuk Aktiviti/ Tingkah laku berikut ...	S1: ya/ tidak	S2: Jika ya, berapa kerap	S3: Jika secara kerap/berkala, berapa kerap	S4: Jika hanya untuk suatu tempoh/seketika, berapa hari semuanya	S5: Kesan terhadap kehidupan seharian	S6: Penyebab (kod jawapan)
c	**Memberus gigi**	1☐ ya<br>2☐ tidak	1☐ secara kerap/ berkala<br>2☐ hanya untuk suatu tempoh/seketika	1☐ kurang daripada sekali dalam sebulan<br>2☐ 1-2 kali dalam sebulan<br>3☐ 1-2 kali dalam seminggu<br>4☐ 3-4 kali dalam seminggu<br>5☐ setiap hari atau hampir setiap hari<br>9☐ tidak pasti	1☐ sehingga 5 hari<br>2☐ sehingga 15 hari<br>3☐ sehingga 30 hari<br>4☐ sehingga 3 bulan<br>5☐ lebih daripada 3 bulan<br>9☐ tidak pasti	0☐ tiada kesan<br>1☐ kesan yang sangat kecil<br>2☐ kesan kecil<br>3☐ kesan sederhana<br>4☐ kesan teruk<br>5☐ kesan yang sangat teruk<br>9☐ tidak pasti	
d	**Menjalankan kerja penting atau peranan masyarakat**	1☐ ya<br>2☐ tidak	1☐ secara kerap/ berkala<br>2☐ hanya untuk suatu tempoh/seketika	1☐ kurang daripada sekali dalam sebulan<br>2☐ 1-2 kali dalam sebulan<br>3☐ 1-2 kali dalam seminggu<br>4☐ 3-4 kali dalam seminggu<br>5☐ setiap hari atau hampir setiap hari<br>9☐ tidak pasti	1☐ sehingga 5 hari<br>2☐ sehingga 15 hari<br>3☐ sehingga 30 hari<br>4☐ sehingga 3 bulan<br>5☐ lebih daripada 3 bulan<br>9☐ tidak pasti	0☐ tiada kesan<br>1☐ kesan yang sangat kecil<br>2☐ kesan kecil<br>3☐ kesan sederhana<br>4☐ kesan teruk<br>5☐ kesan yang sangat teruk<br>9☐ tidak pasti	
e	**Tidur dan bersenang-senang**	1☐ ya<br>2☐ tidak	1☐ secara kerap/ berkala<br>2☐ hanya untuk suatu tempoh/seketika	1☐ kurang daripada sekali dalam sebulan<br>2☐ 1-2 kali dalam sebulan<br>3☐ 1-2 kali dalam seminggu<br>4☐ 3-4 kali dalam seminggu<br>5☐ setiap hari atau hampir setiap hari<br>9☐ tidak pasti	1☐ sehingga 5 hari<br>2☐ sehingga 15 hari<br>3☐ sehingga 30 hari<br>4☐ sehingga 3 bulan<br>5☐ lebih daripada 3 bulan<br>9☐ tidak pasti	0☐ tiada kesan<br>1☐ kesan yang sangat kecil<br>2☐ kesan kecil<br>3☐ kesan sederhana<br>4☐ kesan teruk<br>5☐ kesan yang sangat teruk<br>9☐ tidak pasti	

Sambungan Jadual jawapan…

	Kesukaran untuk Aktiviti/ Tingkah laku berikut ...	S1: ya/ tidak	S2: Jika ya, berapa kerap	S3: Jika secara kerap/berkala, berapa kerap	S4: Jika hanya untuk suatu tempoh/seketika, berapa hari semuanya	S5: Kesan terhadap kehidupan seharian	S6: Penyebab (kod jawapan)
**f**	**Senyum, ketawa dan menunjukkan gigi tanpa rasa malu/segan**	1☐ ya<br>2☐ tidak	1☐ secara kerap/ berkala<br>2☐ hanya untuk suatu tempoh/seketika	1☐ kurang daripada sekali dalam sebulan<br>2☐ 1-2 kali dalam sebulan<br>3☐ 1-2 kali dalam seminggu<br>4☐ 3-4 kali dalam seminggu<br>5☐ setiap hari atau hampir setiap hari<br>9☐ tidak pasti	1☐ sehingga 5 hari<br>2☐ sehingga 15 hari<br>3☐ sehingga 30 hari<br>4☐ sehingga 3 bulan<br>5☐ lebih daripada 3 bulan<br>9☐ tidak pasti	0☐ tiada kesan<br>1☐ kesan yang sangat kecil<br>2☐ kesan kecil<br>3☐ kesan sederhana<br>4☐ kesan teruk<br>5☐ kesan yang sangat teruk<br>9☐ tidak pasti	
**g**	**Mengekalkan keadaan emosi biasa tanpa rasa terganggu**	1☐ ya<br>2☐ tidak	1☐ secara kerap/ berkala<br>2☐ hanya untuk suatu tempoh/seketika	1☐ kurang daripada sekali dalam sebulan<br>2☐ 1-2 kali dalam sebulan<br>3☐ 1-2 kali dalam seminggu<br>4☐ 3-4 kali dalam seminggu<br>5☐ setiap hari atau hampir setiap hari<br>9☐ tidak pasti	1☐ sehingga 5 hari<br>2☐ sehingga 15 hari<br>3☐ sehingga 30 hari<br>4☐ sehingga 3 bulan<br>5☐ lebih daripada 3 bulan<br>9☐ tidak pasti	0☐ tiada kesan<br>1☐ kesan yang sangat kecil<br>2☐ kesan kecil<br>3☐ kesan sederhana<br>4☐ kesan teruk<br>5☐ kesan yang sangat teruk<br>9☐ tidak pasti	
**h**	**Menyukai berinteraksi dengan orang lain**	1☐ ya<br>2☐ tidak	1☐ secara kerap/ berkala<br>2☐ hanya untuk suatu tempoh/seketika	1☐ kurang daripada sekali dalam sebulan<br>2☐ 1-2 kali dalam sebulan<br>3☐ 1-2 kali dalam seminggu<br>4☐ 3-4 kali dalam seminggu<br>5☐ setiap hari atau hampir setiap hari<br>9☐ tidak pasti	1☐ sehingga 5 hari<br>2☐ sehingga 15 hari<br>3☐ sehingga 30 hari<br>4☐ sehingga 3 bulan<br>5☐ lebih daripada 3 bulan<br>9☐ tidak pasti	0☐ tiada kesan<br>1☐ kesan yang sangat kecil<br>2☐ kesan kecil<br>3☐ kesan sederhana<br>4☐ kesan teruk<br>5☐ kesan yang sangat teruk<br>9☐ tidak pasti	
